# Tailored job coaching for people with severe mental illness living in supported housing settings: A realist approach

**DOI:** 10.1371/journal.pone.0343044

**Published:** 2026-03-04

**Authors:** Joep Binkhorst, Josje Dikkers, Roland Blonk, Marc van Veldhoven

**Affiliations:** 1 Research Group Decent Work, Research Centre for Social Innovation, University of Applied Sciences Utrecht, Utrecht, The Netherlands; 2 Department Tranzo, Scientific Center for Care and Wellbeing, Tilburg School of Social and Behavioral Sciences, Tilburg University, Tilburg, The Netherlands; 3 Optentia, North West University, Vanderbijlpark, South Africa; 4 Department Human Resource Studies, Tilburg School of Social and Behavioral Sciences, Tilburg University, Tilburg, The Netherlands; Mugla Sitki Kocman University: Mugla Sitki Kocman Universitesi, TÜRKIYE

## Abstract

**Background:**

For people with severe mental illness (SMI) residing in supported housing settings, finding and maintaining paid or unpaid work is challenging. This study was initiated to examine how professionals tailor job coaching trajectories to effectively address the specific needs of clients. The aim was to unravel the complexity of these trajectories, providing a deeper understanding of how and under what circumstances people with SMI in supported housing can obtain and sustain meaningful daily activities, including paid or unpaid work, as part of their recovery journey.

**Methods:**

Interviews were conducted with 24 clients with severe mental illness (SMI) and their job coaches (N = 15) in dyads. Additionally, two mixed focus group discussions were held with job coaches (n = 16) and their supervisors (n = 2). A realist evaluation approach was used to determine what works for whom, how, and under which conditions. Self-Determination Theory (SDT) served as the analytical framework to explore the motivational factors that drive clients to seek and retain paid or unpaid work.

**Results:**

Our findings are structured in three sections, each focusing on context-intervention-mechanism-outcome (CIMO) configurations. These configurations illustrate how job coaches address clients’ needs for relatedness, competence and autonomy. The findings provide a deeper understanding of the inner workings of job coaching trajectories, showing how job coaches foster autonomous motivation and thereby enable clients to obtain and retain both paid and unpaid work.

**Conclusions:**

This study highlights that there is no universal approach to job coaching. Job coaching requires a tailored approach with a strong emphasis on building personal-professional relationships and adapting interventions to individual circumstances.

## Introduction

This paper provides a comprehensive analysis of how and under what circumstances job coaching trajectories for people with severe mental illness (SMI) work. It presents findings from a realist evaluation study that explores the intricate dynamics of these trajectories. The study highlights that there is no one-size-fits-all approach to job coaching. Successful trajectories are characterized by job coaches who adapt their strategies to the unique circumstances of their clients, addressing their needs for relatedness, competence, and autonomy. This personalized approach fosters autonomous motivation, facilitating clients’ ability to secure and sustain paid or unpaid employment as part of their recovery journey.

Many studies have shown that for people with SMI, paid employment or other forms of participation can positively affect physical and mental well-being and benefit recovery [1–4]. SMI is non diagnostic but refers to individuals who experience symptoms for a longer period of time and have limitations in social and/or societal functioning [5]. It includes people dealing with depression, eating disorders, autism, or addiction. Engaging in paid or unpaid work for people with SMI provides structure to one’s day or week, enhances self-confidence, and fosters personal identity and accomplishment [6–8]. Consequently, this engagement can strengthen their sense of belonging to society [9].

### Effectiveness of job coaching

To support people with SMI to find and keep paid or unpaid work, several effective rehabilitation approaches have been developed [10,11]. Individual Placement and Support (IPS) is widely considered as effective for helping people obtain and maintain paid employment and education [12–15]. Another example, is the Boston Psychiatric Rehabilitation (BPR) intervention [10]. This approach addresses broader goals, such as housing, leisure activities and their social and family network [16].

While these approaches show positive outcomes in obtaining work, evidence of their (long-term) impact on maintaining work, mental health and recovery is less clear. A systematic review [17] reported that the effects on secondary outcomes (e.g., job satisfaction, self-esteem, or the development of socio-economic and mental health outcomes) remain ambiguous. In The Netherlands one study found that IPS increased chances to find competitive employment, but did not improve health related outcomes [18]. A comparison of 22 (systematic) concluded that IPS has no consistent positive or negative effect on quality of life, functioning, or mental health [19]. Similarly, a Dutch RCT study on BPR showed improvements in paid or unpaid work, quality of life, and psychosocial functioning in both the experimental and the control group, suggesting indicating that outcomes are not determined by the specific methodology used [20].

### Paid work is not a panacea

Despite the current attention in both academia and professional practice for supporting people with SMI to secure paid work, studies note that paid work is not without risks. It can also increase stress, anxiety, or feelings of exclusion [21–23]. It has been suggested that paid work should not always be seen as a ‘panacea’ for recovery (24). As underlined: “An important balance [is] to be struck here between the dangers of forcing people back to work and the dangers of excluding them from it through a combination of ignorance, prejudice, and lack of effective help” [24, p. 5]. Furthermore it was found that other forms of participation, besides competitive employment, can also be beneficial for people with SMI. This applies particularly to aspects related to quality of life, I [8]. Research indicated that sheltered work, work in social firms [25–28], or voluntary work [3,29] can also aid the recovery process. Such noncompetitive forms of work should therefore not be overlooked.

Our review of the literature demonstrates that supporting people in finding paid work, or other forms of employment requires a tailored approach. There is no “one-size-fits-all” solution [6, p.2]. Successfully establishing a trajectory depends on several factors. These include: the implementation quality (fidelity) [30,31], the dynamics between a client and a job coach [32,33], and the clients’ capabilities, desired goals, whishes, or motivation [34–36]. Contextual factors also influence the outcome of job coaching trajectories. On a personal level these factors include the client’s health situation (e.g., diagnosis or severity of symptoms), work and skills (e.g., work experience, underqualification, employment gaps) and socio-demographics (age, economic status, etcetera). Other relevant aspects are the social network (support from family and friends), financial situation (the use and type of social benefits, personal assets), and housing situation (stability in residency) [6,37].

The reviewed literature also shows that most studies adopt an RCT design comparing experimental groups to control groups or ‘care as usual’ to assess overall effectiveness. What remains understudied is how, for whom and under which conditions these approaches work. Specifically, there is a gap in understanding how job coaches can tailor their activities to clients’ personal circumstances and sustain motivation throughout the trajectory, thereby enabling them to obtain or maintain paid or unpaid work.

### A realist approach

Researchers suggest Realist Evaluation to better understand the inner workings job coach trajectories, by examining the causal relationships within them. This approach offers a way to complement control-designed RCT studies [38]. Due to the ongoing changes in people’s ‘real life’ situations, we argue that context cannot be kept stable, as is assumed in controlled experiments [39,17]. From a realist perspective, context is seen as a ‘multifaced factor’ that impacts whether intervention mechanisms work as intended [40,41]. A realist evaluation approach makes it possible to investigate how in specific contextual circumstances job coaches intervene in order to foster mechanisms that subsequently result in specific outcomes (i.e., finding or maintaining work or other forms of participation) [42].

### Research aim and setting

Using a realist approach, this study aims to better understand how job coaches tailor their activities to support people with SMI to find paid work or other forms of participation. We do this by investigating the experiences of clients with SMI living in supported housing settings (sheltered and outpatient), job coaches and supervisors. In this setting, job coaches offer support to clients with SMI to obtain several forms of participation (depending on their client’s wishes). This can be directed towards paid work, unpaid work (voluntary work or daytime activities) or education. In these trajectories, job coaches offer support from a recovery oriented approach [43]. When suited and possible, job coaches that are IPS certified establish trajectories based on the IPS principles, for example when specific funding structures or social benefit requirements allow for it [44]. In some trajectories job coaches combined or alternated between these approaches. Job coaches adapt their activities and interventions to the needs of their clients, guided by their professional judgment and discretionary considerations. For the purpose of this study, we focused on the configurations between the contextual factors in which job coaching trajectories takes place, the characteristics of the job coaches activities and the resulting mechanisms that contribute to obtaining and maintaining paid or non-paid work for people with SMI. In this study, variation in context was limited to differences in clients’ personal circumstances, as other contextual factors at the meso- or macro-level cannot be influenced within the scope of a job coaching trajectory.

### Self-determination theory

We articulate the role of motivation as an important element to determine whether a trajectory towards paid or unpaid work becomes successful. Researchers frequently refer to motivation to understand human behavior, as it forms a core component of numerous theoretical frameworks. [45,46]. In this study we use self-determination theory (SDT) as a framework. SDT is a psychological theory of behavior change [47]. To clarify how this theory informs our approach, the following section outlines its theoretical foundations, examines its practical application within recovery-oriented care, and explains how these elements are integrated into this study.

SDT is based on the idea that people want to be ‘autonomous’ or ‘self-determined’, meaning: being able to choose how to satisfy your needs when interacting with others and pursuing activities you inherently enjoy [48,49]. Studies have shown its relevance in the work domain, as it contributes to our understanding of employee wellbeing and work-related behavior [50].

The framework is also considered useful to understand the underlying mechanisms of recovery processes for people with severe mental illness [51]. SDT shows several similarities with recovery-oriented practices for people with long term mental health issues as it is based on almost identical motivational concepts [51]. Following SDT, motivation is stimulated when people experience ‘relatedness’ towards others. From the perspective of delivering recovery oriented care, this implies that professionals focus on people’s ‘entire life’ (mind, body, spirit). They involve significant others (peers, family, colleagues, etc.) in their support. Furthermore, professionals are ‘present’ to help people to feel accepted in society, communities or systems of care [52]. Competence related activities refer to delivering the message of hope towards clients. It also involves adaptation of professionals to the unique strengths, needs, preferences, experiences and cultural backgrounds. This way they help clients to look for challenges that reduce one’s personal suffering [51,52]. The principle of recovery-oriented care emphasizes the importance of autonomy by allowing clients to choose their own path towards recovery. Clients should be able to choose among options and participate in all decisions that affect them. Professionals acknowledge that recovery is nonlinear and that it may involve setbacks. They do this so that clients can learn from these experiences. This also means that clients are responsible for their self-care and journeys of recovery. Professionals align with their clients’ personal life situation, they recognize and stimulate clients to empower and enable them to act in a self-determined manner [51,52].

### SDT in relation to job coaching

Although job coaching for people with SMI and the role of motivation have received considerable attention [35], little is known about how job coaches apply motivational mechanisms in practice. This gap is critical because understanding these mechanisms can explain why some trajectories succeed while others fail across different contexts. To address this, the present study clarifies how motivational mechanisms operate in job coaching trajectories. Specifically, we examine how job coaches in successful trajectories address and acknowledge their clients’ needs for autonomy, competence, and relatedness to help them find or maintain employment. Within a realist framework, SDT identifies these needs as key motivational mechanisms that shape outcomes.

**Fig 1:**
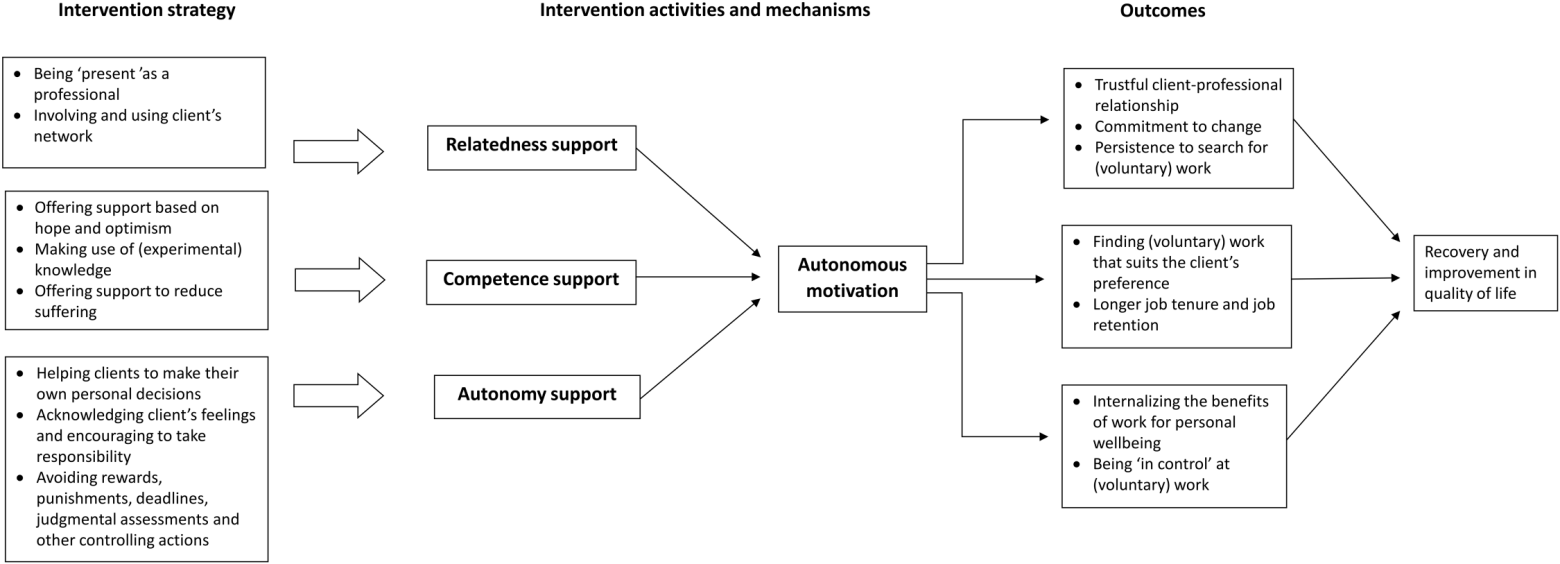
Recovery oriented care in relation to self-determination theory.

We expect that successful trajectories involve job coaches tailoring their intervention activities to the specific personal and contextual circumstances of their clients (e.g., their socio-demographical and general health situation, the amount of support from peers or family or financial situation). We hypothesize that relatedness is fostered through active presence and engagement, competence through encouragement and valuing experiential knowledge, and autonomy through supporting decision-making and avoiding undue pressure. Consequently, we predict that clients who feel autonomously motivated by their job coach are more likely to recognize the benefits of paid or unpaid work for their personal mental well-being and recovery. This, in turn, enhances clients’ perseverance in obtaining or maintaining employment (paid and unpaid) and ultimately contribute to personal recovery and quality of life. Fig 1 schematically represents the reasoning outlined above, illustrating the connection between recovery-oriented care and Self-Determination Theory, and depicting the causal relationship between the intervention strategies, the three psychological needs, and potential outcomes.

By integrating a realist approach with SDT, this study contributes to the literature by uncovering how and why job coaching strategies work in different conditions. This provides a deeper understanding of the mechanisms behind successful job coaching trajectories and offers practical insights for designing more effective, recovery-oriented interventions.

## Methods

### Study design

This qualitative study followed the realist evaluation methodology with a focus on the role of motivation as an important mechanism in job coach trajectories. The study took place as part of a practice-oriented research project in collaboration with a supported housing (sheltered and outpatient) organization in Utrecht, he Netherlands.

Realist evaluation is considered a particularly suitable research method as this method focusses on the complexity of factors that influence the effects of social health care interventions such as job coach trajectories [37]. The premise behind realist methodology is that programs (or interventions) work differently in different contexts. This means that an intervention (e.g., a job coaching approach) that is ‘successful’ in one context may ‘fail’ in another, because the mechanisms needed for success are triggered to different degrees in different contexts [53]. From a realist evaluation perspective, interventions may enable specific mechanisms and outcomes subsequently. Mechanisms can be perceived as the ‘underlying causal processes’ that generate behavior [54]. Mechanisms are often not directly observable, but they are still considered ‘real’ (e.g., ‘trust’ in the relationship between a client and job coach) [55]. They exist within the cognitive or emotional reasoning of those who are the subject of a specific intervention and are triggered or not depending on the contextual circumstances. In realist evaluations the question asked is not ‘does the intervention work?’ but ‘what works, for whom, under what conditions and how?’

In this study we followed the context-intervention-mechanism-outcome (CIMO) logic [56], that stems from the concept of realist evaluation [57,58]. Box 1 offers the definition of realist terminology [59]. The unit of analysis in this study was the job coach trajectory itself. Context was defined as the differences in the personal circumstances that may influence how an intervention works, such as a client’s age, diagnosis or prior work experience. For example, a client with limited work experience may require more structured guidance than one with extensive experience. The intervention was defined as the actions and behavior of job coaches in interactions with clients such as weekly meetings and collaborative development of an individualized support plan. Mechanisms are the underlying responses and reasoning processes triggered from this. For instance, experiencing competence through positive feedback. Outcomes refer to the results of these processes, such as taking steps towards employment or engaging in work. In this case steps to find or maintain work).

**Box 1. Definitions of realist terminology**

**Definitions of realist terminology**

Middle range theories: These theories bridge the gap between specific working hypotheses used to design and evaluate interventions, and broad, systematic theories that explain general patterns in social behavior, organization, and change. They are abstract enough to offer explanatory power, yet concrete enough to be tested and applied in practice. [60].

Context: This refers to to the backdrop of an intervention. It includes enabling or disabling aspects beyond the intervention itself that shape the activities carried out [60,61] Context in this study refers to the individual circumstances, capacities and characteristics of clients [62]. Contextual factors at the meso level (e.g., caseload size, organizational characteristics) and macro level (e.g., cultural aspects, unemployment rates) are not taken into account.

Intervention: These are purposeful actions or measures (products, processes, services or activities) that are to solve a problem or need, and to influence outcomes [63].

Mechanisms: These are triggered by the intervention within a given context [64]. They refer to underlying entities, processes or structures that influence the outcome [54], including participants’ resources and their cognitive and emotional responses to the intervention or exposure.

Outcome: This refers to what can be measured in terms of impact across the target population, using measurable or measured indicators. Outcomes can be considered as quantitative or qualitative and intended or unintended [65].

### Data collection and procedures

Clients were recruited based on (stratified) purposive and theoretical sampling [66] between April 1 and May 31, 2022. We selected clients who had already obtained paid or unpaid work and those still searching. To achieve a heterogenous sample, we focused on four case types [66, p. 50]: unique cases (e.g., clients with unusual circumstances, such as a severe illness combined with strong social resources), critical cases (e.g., clients meeting the conditions for a recovery-oriented intervention and illustrating whether key mechanisms operate as expected), extreme cases (e.g., clients who, despite severe symptoms, were able to find or maintain employment), and typical cases (e.g., clients with common diagnoses who follow standard job-coaching trajectories to secure and maintain work). Job coaches made a first selection of clients based on these criteria; the research team decided on the final research sample. Table 1 provides an overview of the included clients and their characteristics. Clients did not receive vocational support from other professionals. However, clients with paid or unpaid work often received support from an internal job coach or supervisor. All participants gave informed consent.

**Table 1:** Client characteristics.

#	Gender	Diagnosis/ Mental vulnerabilities^a^	Other characteristics	Benefits type	Participation goal
**1**	Male	Depression, Substance abuse disorder, PTSD	Financial problems, Debts	None	Paid Work
**2**	Female	Depression, Anxiety disorder	–	Social assistance benefits	Paid work
**3**	Male	Psychotic Disorder, Asperger’s syndrome, PTSD, Substance use disorder	Financial problems, Debts	Wajong benefit (Disablement Assistance Act for Handicapped Young Persons)	Education/ Voluntary work
**4**	Male	Self-neglect	–	Social assistance benefits	Voluntary work
**5**	Male	Attention Deficit disorder, Psychosis	Financial problems	Wajong benefit (Disablement Assistance Act for Handicapped Young Persons)	Voluntary work
**6**	Male	Autism spectrum disorder	–	Social Benefits	Start education towards paid work
**7**	Male	Schizophrenia	Physical illness, history in forensic psychiatric treatment clinic	Disablement Benefits Act	Paid work
**8**	Male	Autism spectrum disorder, Psychotic Disorder	–	Work and Income According to Labour Capacity Act	Paid work
**9**	Female	Asperger’s syndrome	Cohabiting	Unemployment Benefit Act	Paid work
**10**	Male	Autism spectrum disorder	–	Wajong benefit (Disablement Assistance Act for Handicapped Young Persons)	Paid work
**11**	Male	Autism spectrum disorder	–	Wajong benefit (Disablement Assistance Act for Handicapped Young Persons)	Paid work
**12**	Male	Complex PTSD	Job agreement target group, Financial problems, Debts	Social assistance benefits	Paid work
**13**	Male	Bipolar disorder	Financial problems	Work and Income According to Labour Capacity Act	Day activities
**14**	Female	PTSD	Financial problems, Debts	Social assistance benefits + internship compensation	Education
**15**	Female	Asperger’s syndrome	–	Social assistance benefits	No activity
**16**	Female	Autism spectrum disorder	Job agreement target group	None	Paid work
**17**	Female	Bipolar disorder, ADHD	–	Social assistance benefits	Paid work
**18**	Male	Autism spectrum disorder, Substance use disorder	–	Social assistance benefits	Voluntary work
**19**	Female	Autism spectrum disorder, Eating disorder	–	None	Voluntary work
**20**	Male	Psychosis, Bipolar disorder	Self-neglect	Work and Income According to Labour Capacity Act	Voluntary work
**21**	Male	Autism spectrum disorder	Job agreement target group	None	Paid work
**22**	Male	Substance use disorder	No residence permit, housing insecurity	None	No activity
**23**	Male	Substance use disorder	Formely homeless	Social assistance benefits	Paid work
**24**	Male	Depression, Substance use disorder	Formely homeless	Social assistance benefits	No activity

^a^As indicated by clients themselves.

Data collection involved dyadic interviews followed by individual client interviews. Thereafter, two focus groups were subsequently organized to refine insights from the interviews.

#### Interviews.

We conducted dyadic interviews with clients (n = 24) and their job coach (n = 15), followed by individual interviews with clients (n = 24). The dyadic interviews had a duration of 45–90 minutes. The follow-up interviews had a duration of 30–60 minutes. All interviews took place between May 13 and August 25 2022. For the dyadic interviews we used a realist interviewing technique referred to as the ‘teacher/learner cycle’ [67–69]. This implies that the interviewer and interviewee ‘teach’ each other about the inner workings (‘theories’) of the intervention that is being explored. In this way, the realist interview is a method for data collection to ‘confirm, refute or refine’ theories about how the intervention works [68,69]. Example question: ‘*Some research shows that being able to make your own decisions in a trajectory, contributes to finding a suitable job. Is this something you recognize? Can you explain?’* Conducting interviews in dyads allowed clients and their job coach to jointly reflect on what worked during the trajectory, how and under what conditions. Follow-up interviews gave clients space to elaborate on issues they felt were missed during the dyadic interview or that they did not feel comfortable discussing in the presence of their job coach. CIMO logic was used to guide both the dyadic and follow-up interviews, ensuring that questions elicited information about the specific context in which the job-coach trajectory took place, the characteristics of the intervention, and the underlying mechanisms influencing the outcomes.

#### Focus groups.

Following the interviews, we conducted two focus groups with job coaches and their supervisors, each lasting two hours, to reflect on the interview findings. The first focus group included 7 job coaches and one supervisor; the second, 9 job coaches and one supervisor. Sessions took place at the Utrecht University of Applies Sciences in January and February 2023. Each meeting began with an introduction to the study and an opportunity for questions. An example question for the participants was: *‘Can you explain what it is about proximity of a job coach to his or her client that contributes to finding a job or keeping a job?’* Discussions started with a list of topics derived from the dyad interviews that participants could prioritize. Based on the prioritization, initial CIMO configurations [56] were presented for participants to confirm, refine or refute using their own experiences.

#### Ethical considerations.

This study was performed in line with the principles of the Declaration of Helsinki. Prior to the study, the study proposal was reviewed and approved by the Ethics Review Board of Tilburg University – Tilburg School of Social and Behavioral Sciences (number TSB_RP265). All participants were informed verbally and in writing about the study before signing a written informed consent form. Clients received a €20,- voucher to acknowledge their contributions.

#### Data analysis.

Data analysis took place based on the integrated insights from both the interviews and focus groups. First, the dyadic interviews and focus group discussions were audiotaped and transcribed verbatim. Reports were made for all audiotaped follow-up interviews. Next, a thematic analysis strategy [70] was employed using Atlas.ti version 23.0.6.0. After initial coding, codes were sorted into potential themes. Two researchers then identified relationships between codes to create initial CIMO configurations. Reflections from the focus group meetings were used to further refine the initial CIMO configurations. Finally, based on Self-Determination Theory, codes were clustered and discussed with a third researcher until consensus was reached.

To meet rigor criteria [71] the authors of this paper interpreted and analyzed the data together. Interviewees did not provide feedback on the findings. However, the preliminary insights were submitted to professionals and policy makers in two focus group meetings. We used the RAMESES II reporting standards for realist evaluations [53] and COREQ for qualitative research [72].

## Results

The study findings are organized into three sections. Using Self-Determination Theory (SDT) as a middle-range theory, we explored how job coaches address the basic human needs for relatedness, autonomy, and competence to motivate their clients to find or maintain paid or unpaid work. Each section outlines the contextual backdrop of the trajectory. This refers to the personal circumstances of clients that job coaches need to consider. It then describes the activities of job coaches as part of the intervention. Next, it highlights the underlying mechanisms these activities trigger, which in turn evoke client responses or reasoning (in bold). Finally it presents the subsequent outcomes.

### Relatedness

#### Context.

Loneliness is a significant problem among people with a severe mental illness. Taking steps towards work or other forms of participation is complex and depends on several personal circumstances (e.g., social network, health or housing situation). The idea to take these steps on your own can feel overwhelming. Clients in this study expressed a clear need for support to overcome this challenge.

#### Intervention activities.

In the trajectories studied, building a relationship between professionals and clients emerged as crucial. This starts by connecting with clients on a personal level. Job coaches create common ground through informal often non-work-related activities (e.g., small talk, walks, cardio exercises). Such aholistic approach is essential for building trust. Job coaches tailor their actions by considering factors like age, mental health, family support, and financial situation.

Home visits allow job coaches to better understand personal issues. These visits help identify wellbeing signals early, prevent relapse, and promote job retention. Most clients appreciate this connection to other life domains, such as healthcare, housing, and general wellbeing. One client explains:

*“They are just more engaged and nearby, also in my daily activities. [My job coach] is more involved with this than my supporters at work. When I call in sick my job coach knows that there might be other things going on in my life. (…) [My job coach] focuses on what I need as a person, not just what I need to re-integrate in work. It’s often about my work-life balance.” [R14]*

Another key activity of job coaches is acting as a liaison between their client and other stakeholders, such as municipalities, the Employee Insurance Agency, other institutions, and potential employers. They make phone calls, visit job markets, arrange interviews, and advice on workload capacity.

#### Mechanisms.

These intervention activities support mechanisms that foster autonomous motivation. First, they help clients **feel ‘equivalent’** to their job coach, gaining trust and enabling them to **internalize the job search process**. Second, clients emphasize the importance of **proximity and approachability** of job coaches. Without this closeness, they say they would not have achieved their current progress in terms of work and mental health. A job coach illustrates:

*“I have a client who is working for three years. So everything runs smoothly, but then her dad dies, mom is getting ill. (…) So these life events have a major impact. At moments like these [the employer] refers to me because she already trusts me. I then can help [my client] with communication for instance towards the organization where she works, so that also in this situation she remains able to function at the workplace.” [Focus Group 2]*

Clients note that interactions with other professionals, such as re-integration professionals, occupational specialists, job coaches from the Employee Insurance Agency (UWV) or municipalities are often volatile, making it harder to accept advice. One client shares:

*“I have no good experiences with [the Employee Insurance Agency]. I don’t trust them, at least not the people I talked to. (…) I had an assessment interview with one person, reading my dossier and saying: ‘You do not have a depression, you do not look like that’.” [R7]*

With the support of a job coach as a spokesperson, such encounters become less burdensome. A third mechanism, therefore, relates to **empowering** clients to **interact with institutions** to facilitate dealing with complex laws and regulations.

#### Outcomes.

These mechanisms lead to several outcomes. On an interpersonal level, they help to build a *trustful relationship* between job coaches and clients. On a personal level, they strenghen the client’s *commitment* to searching for work or other forms of participation. The experienced egalitarian relationship and proximity of a job coach provide space for reflection on suitable activities and reinforce perseverance. Addressing the need for relatedness also increases clients’ *trust in institutions* and helps overcome negative past experiences.

In summary, mechanisms that strengthen relatedness, such as building an egalitarian relationship, maintaining proximity and approachability, and supporting clients in interactions with institutions, lead to outcomes including a trustful relationship with the job coach, greater commitment to searching for work or other forms of participation, strengthened perseverance, and increased trust in institutions.

### Competence

#### Context.

Several clients in this study report difficulties articulating their competencies. They often feel underqualified or believe they lack work experience, which undermines confidence in achieving their desired goals. Accepting support in these situations is challenging. Different approaches are required based on age or educational level. Financial or housing circumstances and social networks can also influence the ability to articulate their competencies, often due to stigma.

#### Intervention activities.

Job coaches’ competence-related activities involve finding challenges that match clients’ capabilities. They are persistent and proactive in creating work opportunities, such as finding vacancies or helping clients with social benefit applications. Their goal is to help clients find a form of participation (either paid or unpaid) that aligns with their skills, interests, and wishes, considering educational level, work experience, and personal circumstances (e.g., general health status, severity of symptoms).

When a suitable employment setting is found, job coaches support clients with job interviews and writing motivational letters. They explore working conditions together, discussing hours, breaks, and workplace atmosphere. They also address relapse prevention by identifying triggers and, with client consent, sharing these with managers. Job coaches assess the implications of employment for social benefits and financial situations, adapting support to match clients’ capabilities:

*“I really adapt to what is needed. For you, doing a job interview was not a problem: you could do this on your own, with me only in the background involved (although we do talk it through up front or afterwards). But writing a motivational letter was much more difficult for you. So: we write a letter together.” [job coach about R14]*

When a job is secured, support shifts to job retention. The focus then is on workload, work-life balance, setting personal boundaries, and seeking help when needed. Some job coaches visit clients at the workplace, arranging meetings with managers to create a ‘collective memory’ about mental vulnerabilities. Others discuss support roles with HR or provide remote support via calls or messages. Staying close to clients becomes harder as job tenure increases.

#### Mechanisms.

These competence supporting activities help clients recognize and acknowledge their capabilities. Professionals demonstrate professional restraint, ensuring they don’t take over too much. This approach strenghtens **resilient to setbacks** and **dealing with stigma** related to mental vulnerabilities. Furthermore, job coaches **provide positive feedback** to help clients **feel more competent in their achievements,** even when a contract is not being renewed or in case of a relapse. Job coaches also help clients **recognize their ‘added value’** in the workplace and encourage them to **embrace their vulnerabilities** as part of their identity:

*“If they want to know what is wrong with me, they can just ask and I’ll answer so they have an image of who I am so they can take this into account. This might prevent having a psychosis or when I’m having a psychosis, they can help.” [R5]*

Another mechanism involves supporting clients in **defining wishes, hopes, and expectations** to allign support to their clients’ competences, values and interests. This helps clients **broaden their horizons, focus** and **envision themselves** in work-related situations. This **provides a sense of control** about their possibilities strengthening motivation to search for and apply for jobs. Articulating hopes and dreams accelerates the job search process:

*“I really feel they take [my dreams and wishes] seriously. For me dreams and passion are very important. Right now, thinking about it, I feel it too: I really live up. I think the importance of emphasizing my dreams may even be bigger than they think.” [R19]*

Using clients’ expectations, dreams, and wishes helps them **feel competent and empowered** to achieve personal goals. For those struggling to define these aspirations, goal attainment is more difficult:

*“Sometimes I just think about [my clients]: ‘Oh wow, you are missing any perspective of where you want to go, whatever it may be’. And then he starts crying and he says: ‘I’m missing any direction of where I’m going. I have all kinds of therapy but no idea of where I’m going.” [Job coach in focus group 1]*

#### Outcomes.

Intervention activities addressing clients’ need for competence activate mechanisms enabling them to pursue goals aligned with their values and interests, in both *paid and unpaid work*. Clients underline that both can appeal to their need for competence. Nevertheless, most rank paid work higher due to its social status. Voluntary work is seen as “helping” or providing “structure” in life. “Paid work [on the other hand] really asks something from you, you really need to take it seriously. That is a real responsibility.” Several clients therefore indicate that to avoid high work pressure or demanding paid work settings, they tend towards trajectories focusing on finding voluntary work. Others prefer voluntary work to avoid high pressure, noting it can boost recovery:

*“I was nervous [to start my voluntary work], but when doing so, you experience things that make you feel strong, gives you confidence, security and a sense of belonging. You recognize your own qualities, you receive compliments… All kinds of things you wouldn’t experience when just sitting at home.” [R14]*

In summary, mechanisms that strengthen competence involve helping clients recognize their capabilities, providing positive feedback on achievements, and supporting them in defining hopes and expectations. These mechanisms foster a sense of control and confidence, which in turn lead to outcomes such as pursuing goals aligned with personal values and interests, greater resilience to setbacks and stigma, and increased engagement in work trajectories, whether paid or voluntary.

### Autonomy

#### Context.

Clients often lack (financial) autonomy and self-direction, and perceive past care as controlling. Making their own choices is crucial, especially since trajectories can be lengthy due to changes in (mental) health or other live events, such as moving from a sheltered to outpatient setting. Such events make it harder for a client to focus on (finding) work.

#### Intervention activities.

The findings show that for people with SMI the road to suited (voluntary) work can be long. This is often due to changes in (mental) health situation during the trajectory, or transitions from sheltered to outpatient settings. Such circumstances require tailored trajectories that address the client’s needs at that moment.. Support extends beyond the work domain, with (mental) wellbeing considered most important. Job coaches view paid or unpaid work as a means in the recovery process, not as a goal in itself. Both clients and job coaches explain that rushing towards paid or unpaid work can hinder the alignment between support, capabilities, and personal preferences. Allowing more time during a trajectory enables s job coaches to fulfill clients’ basic psychological need for autonomy.

Job coaches argue that interventions such as IPS, that solely focus on finding and keeping paid work can help achieve clients’ aspired goals. However, they emphasize that a broader approach is often important due to the complexity of the mental health. Even when clients aspire to paid work, job coaches integrate other rehabilitation goals, such as stability in health, social contacts, and housinge. Some trajectories may take years before IPS becomes relevant. As one job coach explains:

*“I have trajectories that take two or three years, just before we are talking about paid work. (…) So there is a whole trajectory that before paid work comes in the picture. (…) therefore I often choose to not go through all the paperwork [for an IPS request]. Because: I always work as an IPS’er. I’m a certified supported employment guy but belief me, how I work goes way deeper than that whole eight principe approach with IPS. I mean, it’s nice, but it is not a silver bullet.” [Job coach about R21]*

#### Mechanisms.

Clients value the tailored approach of their job coach, which allows them to make decisions at their own pace and guide their trajectory. Job coaches note that longer duration gives clients time to **reflect on their mental vulnerabilities**. When clients struggle to acknowledge or address these vulnerabilities, job search becomes more difficult. As an autonomy-related mechanism, job coaches therefore aim to **increase self-insight** by empowering clients to **use their strengths and vulnerabilities.** This approach helps clients not only to determine the type of work they seek, but also enables them to steer and influence the kind of work they engage in and the support they require for job retention. This process enhances their sense of independence. Without self-insight, accepting support is difficult. A client explains:

*“I just wasn’t ready. I just knew. In that situation you don’t accept any support. Now I’m more aware of this, so I think: I face these problems and I want to change this, although I know this is difficult for me. This means that when we are going to look for a job, I know we have to start at a slow pace as I have some self-image issues that we need to deal with.” [R21]*

Job coaches also encourage clients to **take responsibility** on the direction of their trajectory. Clients acknowledge that **promoting self-direction** by job coaches, strengthened their ability to search for (voluntary) work. One client elaborated how this helped her process:

*“I literally said to this man [a former mental health practitioner]: that is not going to happen to me. I’m not going to wallow in my own diagnosis. I just thought: ‘What can I do?’ Instead of dwelling on what I cannot do.” [R9]*

#### Outcomes.

The mechanisms addressing autonomy lead to several outcomes. They help clients internalize the idea that paid or unpaid work can benefit personal growth, social contacts, and financial independence. Without this understanding, the likelihood of dropping out of a trajectory increases. By focusing on autonomy, job coaches support clients in weighing the benefits of voluntary work versus paid work. Paid work can feel too demanding or financially unrewarding. In such cases, voluntary work is attractive for the autonomy it provides (the ability to “be in control” in the workplace). However, a significant disadvantage of volunteering, as noted by clients, is its minimal impact on their financial situation. Clients and job coaches emphasize that the complexity of the Dutch social security system is a major obstacle, as many clients cannot earn more than their social benefits income. This is experienced as frustrating and demotivates clients from securing paid work. As one client elaborates:*“I’m thinking what do I gain from working? In practice working for me does not pay off. I’m not making any more money than someone who just doesn’t do anything all day.” [Emotional and in tears] [R15]*

Summarizing, mechanisms that foster autonomy are enhancing clients’ self-insight, promoting self-direction, and allowing decision-making at their own pace. These mechanisms lead to outcomes such as internalization of the benefits of work for personal growth and independence, informed choices between paid and voluntary employment, and sustained motivation despite structural barriers.

## Discussion

### Summary of findings

This study shows how job coaches can tailor their activities to address clients’ needs for autonomy, competence, and relatedness, thereby activating motivational mechanisms that support people with severe mental illness in finding and maintaining work. To gain insight into these inner workings we conducted a realist evaluation. We applied CIMO logic [56] to explore the configurations between personal circumstances in which job coaching trajectories take place (context), the characteristics of the job coaches’ activities (intervention), the resulting client responses and reasoning (mechanisms) that motivate clients to find and keep paid or unpaid work for people with SMI (outcome).

We focused on motivation as a key concept for determining whether a trajectory towards paid or unpaid work becomes successful. As expected, depending on a client’s s needs and circumstances (context), job coaches must tailor their activities to trigger specific mechanisms. In line with self-determination theory [47], our findings underline the importance of autonomous motivation during job coaching trajectories. Appealing to intrinsic motivation can be considered the ‘generative causal force’ that is important for a trajectory to be successful. Using SDT as a framework enabled us to analyze how job coaches address and acknowledge their clients’ needs for autonomy, competence, and relatedness to help them find or maintain employment. Despite these efforts and the overall satisfaction of clients with the support they received, not every trajectory we studied was successful. Some trajectories showed limited progress, or did not result in obtaining a suitable job.

According to our data, mechanisms that address clients’ need for relatedness are realized by building an egalitarian relationship with clients and staying nearby during the trajectory. Competence is supported through helping clients to deal with setbacks and using their hopes and dreams for goal attainment. Mechanisms that address the need for autonomy are realized when job coaches support clients to increase their self-insight and by promoting self-direction. These mechanisms helped clients reach intended goals, such as finding paid or voluntary work that fits their skills and interests, and making progress in their health and recovery.

In addition to our expectations, our findings illustrate that job coaches continuously shift between or simultaneously address these different needs to keep clients motivated and persevering. For instance, they stay nearby to clients (addressing the need for relatedness) while promoting clients’ self-direction (addressing the need for autonomy). They empower clients to engage in situations where they can show what they’re capable of (competence), support them to make their own choices (autonomy), and help maintain meaningful connections throughout the process (relatedness).

### Comparison with existing literature

The findings underline the importance of investing in a solid working alliance between professionals and their clients, as several other scholars also put forward [32,73,74]. This means that, throughout the trajectory, job coaches aim to establish an egalitarian relationship with their clients. This involves building a personal-professional relationship in which clients feel ‘seen’ and at ease [74]. Achieving this requires attention to topics beyond work-related issues. By doing so, job coaches can build trust, creating space for clients to develop, become stronger and increase their autonomy (ibid). Furthermore, our findings align with mental healthcare research that highlights the importance of self-management to empower individuals [75]. In this regard, the benefits of psycho-education were mentioned. These aspects are necessary to become receptive for support. To internalize support, clients must experience the trajectory as their own individual process, that cannot not be imposed by one another.

Our findings also support earlier research showing that paid work can be both beneficial and challenging for people living with severe mental illness [24]. Consistent with a recent realist synthesis [37] and a systematic mixed-methods umbrella review [6], we identified personal circumstances as crucial elements for job coaches to consider. These include a a client’s health situation (e.g., diagnosis or severity of symptoms), work and skills factors (e.g., work experience, underqualification, employment gaps), socio-demographics (age, economic status, etc.), social network (support from family and friends), financial situation (the use and type of social benefits, personal assets), and housing situation (stability in residency). Additionally, our results reflect the potential disincentive effects of the Dutch welfare system, which several scholars argue can discourage people from seeking paid work (known as the ‘benefit trap’) [7,14].

Taken together, the findings suggest that while IPS has been extensively studied and promoted, its role should be considered in a more nuanced way, as job coaching trajectories often require broader and more individualized forms of support.

### Practical implications

This study focused on tailoring support towards paid work and other forms of participation. We found that, even in trajectories focused on finding paid work, IPS was used in only a small number of cases. This is notable because several job coaches in this study are IPS certified and the organization where our fieldwork took place has the highest IPS fidelity scores. Professionals tended to view IPS as an addition to their ‘toolbox’ at the end of the job coach trajectory. This may be due to the extensive and challenging recovery process. Participants often moved back and forth between the contemplation, preparation, and action stages of recovery [76]. Because of this precariousness, several participants (both clients and professionals) indicated they preferred voluntary work over paid work, as stepping stone towards paid work.

In every trajectory we studied, various life events, regarding health, housing or financial issues, affected the actions and attitudes of both clients and job coaches. These changes often altered the type of support needed. Maintaining continuity in the professional-client relationship is therefore essential for anticipating suchs shifts. Job coaches also need to understand that trajectories for people in supported housing settings often require a significant amount of time before they can be successfully completed. Professionals therefore need adequate training to guide clients through potential relapses or obstacles.

The support provided by the job coaches in this study resembled the Boston Psychiatric Rehabilitation Approach (BPR) BPR has a broader scope than IPS, considering outcomes beyond work, such as housing, education, and social networks [16]. It is suitable for people who are not (yet) sure if they want paid work, or for clients are convinced they are better off starting with voluntary work. Our data demonstrate the complementarity of BPR and IPS. This is also emphasized by scholars who describe IPS as a “framework of methodological and organizational preconditions,” while BPR provides “conversation techniques to support individual processes” [77, p. 231]. Both IPS and BPR begin with the clients’ wishes and stress the importance of setting goals before identifying the necessary skills and competencies to achieve those goals [78].

Despite the expected complementarity, research on integrating IPS and BPR is limited. Most studies look at ways to expand IPS, for example by adding skills training, cognitive support, workplace accommodations, or extra help from peers or family [26]. Combining BPR and IPS emphasizes the role and attitudes of professionals and their relationship with clients, which may lead to improved outcomes [79].

### Strengths and limitations

This study has both strengths and limitations. The first strength is that this study, using realist research methodologies, provided complementary insights to understand the underlying contextual factors and mechanisms leading to the intended outcomes in job coach trajectories for people living in supported housing settings. A second strength lies in the the integration of recovery-oriented practice with SDT, offering a theoretically grounded lens to examine motivational processes. Another strength is the direct involvement of clients, job coaches, and supervisors reflecting a multi-stakeholder approach. Finally, this study is characterized by a substantial amount of interviews, follow-up conversations and focus groups that provided clarity and granulation to make generative causal statements.

There are several limitations to this study. The first limitation is that the job coaches signed themselves up to participate in this study. They subsequently suggested clients to participate. This may have resulted in a selection bias, for instance, when job coaches put their ‘successful’ trajectories to the fore. To avoid this, professionals were instructed to select both ‘successful’ trajectories, as well as trajectories in which realizing the intended outcomes was difficult or not realized. The research team reviewed and discussed the selected cases, in order to create a purposive sample, that reflects the heterogeneity of the clients based on age, gender, care type and focus (towards voluntary or paid work). As a result, we have no reason to assume that the reflections in this study differ from other samples. This, since even the participant’s reflections on ‘successful’ trajectories show the complexity of realizing these outcomes.

Another limitation of this study is that it may have been hard for clients to individually reflect on all interview topics. In order to receive as much information during the interviews as possible, we conducted the interviews in dyads, with clients and their job coach. The goal of a dyadic interview is getting participants to talk about topics in such a way that relevant data is generated [80]. Dyad interviews gave clients and job coaches the opportunity to reflect on the trajectory together and complement each other during the interview. This approach is suitable for this study because the trajectories can be perceived as recovery-oriented practices that assume an egalitarian relationship between professionals and clients [81]. Another effort to avoid that presenting theories was too abstract, the topics were – where necessary – introduced by a more general discussion that did not directly focus on theory testing or refinement. The researcher then asked more generally about the clients experiences with the intervention or other related topics [82,83]. To mitigate effects from power imbalances, as a result of conducting interviews in dyads, clients who felt uncomfortable discussing specific topics during the dyadic interview, were provided the opportunity to come back to this (in absence of their job coach) in the follow-up interviews. In this study the unit of analysis were the VR trajectories themselves. This means that mechanisms outside of the trajectories themselves (e.g., within the workplace) were disregarded.

### Implications for future research

This study provided deeper insights into the complexity of job coaching trajectories by examining context, intervention, mechanisms, and outcomes (CIMO) to explain what works, why, and how for people living in supported housing settings [56,84]. As aforementioned, much attention in the field goes to IPS. This is well-justified, and moreover it is good that supported employment is gaining a stronger foundation in practice. Nevertheless, this study investigated what worked, for whom, and how. From this, we found that needs for people with severe mental illness living in a supported housing setting are often broader than just the ‘work’ domain. We propose that more research is needed to better understand the benefits of integrating IPS with other rehabilitation approaches, such as BPR. Additionally, it would be worthwhile to investigate how the core principles of rehabilitation [85] are applied and recognized by professionals who follow the main components of IPS. Although IPS is based on these rehabilitation principles, its focus on ‘paid work’ may make the connection between IPS and other rehabilitation principles too implicit.

The results of this study illustrate that in the Netherlands, having a paid job often does not ‘pay off’ financially, as clients’ work capacities frequently fall short of earning more than the social benefits level (see the aforementioned ‘benefits trap’). More research is needed to better understand how this type of welfare system influences job coaching interventions and to develop strategies to overcome these financial barriers. Additionally, our findings highlight that the opportunity for clients to make autonomous or self-determined decisions during their trajectory, based on a long-term relationship with professionals, is the most crucial factor for successfully completing a trajectory. This aspect, more than a specific ‘evidence-based’ methodological approach, may be key to tailoring a job coaching trajectory. Future research should further explore these suggestions.

## Conclusions

This study used a realist evaluation design to explore job coaching trajectories for individuals with severe mental illness (SMI) providing a deeper understanding of the interplay between context, intervention, mechanisms, and outcomes. The findings highlight that there is no one-size-fits-all approach for job coaching. Successful job coaching requires tailoring activities to clients’ personal circumstances, addressing their needs for relatedness, competence, and autonomy. This personalized approach fosters autonomous motivation, crucial for obtaining and maintaining work as part of the recovery process.
